# Burden of cancer in the Eastern Mediterranean Region, 2005–2015: findings from the Global Burden of Disease 2015 Study

**DOI:** 10.1007/s00038-017-0999-9

**Published:** 2017-08-03

**Authors:** GBD 2015 Eastern Mediterranean Region Cancer Collaborators, Ubai Alsharif, Charbel El Bcheraoui, Ibrahim Khalil, Raghid Charara, Maziar Moradi-Lakeh, Ashkan Afshin, Michael Collison, Adrienne Chew, Kristopher J. Krohn, Farah Daoud, Daniel Dicker, Kyle J. Foreman, Joseph Frostad, Nicholas J. Kassebaum, Michael Kutz, Haidong Wang, Gebre Yitayih Abyu, Isaac Akinkunmi Adedeji, Aliasghar Ahmad Kiadaliri, Muktar Beshir Ahmed, Ayman Al-Eyadhy, Khurshid Alam, Deena Alasfoor, Raghib Ali, Reza Alizadeh-Navaei, Rajaa Al-Raddadi, Khalid A. Altirkawi, Nelson Alvis-Guzman, Erfan Amini, Nahla Anber, Palwasha Anwari, Al Artaman, Solomon Weldegebreal Asgedom, Tesfay Mehari Atey, Ashish Awasthi, Huda Omer Ba Saleem, Umar Bacha, Aleksandra Barac, Neeraj Bedi, Zulfiqar A. Bhutta, Zahid A. Butt, Carlos A. Castañeda-Orjuela, Abdulaal A. Chitheer, Hadi Danawi, José das Neves, Dragos V. Davitoiu, Subhojit Dey, Samath D. Dharmaratne, Shirin Djalalinia, Huyen Phuc Do, Manisha Dubey, Hedyeh Ebrahimi, Donatus U. Ekwueme, Aman Yesuf Endries, Babak Eshrati, Alireza Esteghamati, Maryam S. Farvid, Seyed-Mohammad Fereshtehnejad, Florian Fischer, Tsegaye Tewelde Gebrehiwot, Sameer Vali Gopalani, Nima Hafezi-Nejad, Randah Ribhi Hamadeh, Samer Hamidi, Habtamu Abera Hareri, Roderick J. Hay, Nobuyuki Horita, Mohamed Hsairi, Mihajlo B. Jakovljevic, Jost B. Jonas, Amir Kasaeian, Nigussie Assefa Kassaw, Yousef Saleh Khader, Ejaz Ahmad Khan, Gulfaraz Khan, Daniel Kim, Yohannes Kinfu, Heidi J. Larson, Asma Abdul Latif, Shai Linn, Raimundas Lunevicius, Hassan Magdy Abd El Razek, Mohammed Magdy Abd El Razek, Azeem Majeed, Reza Malekzadeh, Deborah Carvalho Malta, Desalegn Markos, Peter Memiah, Ziad A. Memish, Walter Mendoza, Tuomo J. Meretoja, Ted R. Miller, Shafiu Mohammed, Vinay Nangia, Quyen Le Nguyen, Trang Huyen Nguyen, Felix Akpojene Ogbo, P. A. Mahesh, Eun-Kee Park, Tejas Patel, David M. Pereira, Farhad Pishgar, Farshad Pourmalek, Mostafa Qorbani, Amir Radfar, Anwar Rafay, Vafa Rahimi-Movaghar, Rajesh Kumar Rai, Saleem M. Rana, Salman Rawaf, Andre M. N. Renzaho, Satar Rezaei, Kedir Teji Roba, Gholamreza Roshandel, Mahdi Safdarian, Sare Safi, Saeid Safiri, Payman Salamati, Abdallah M. Samy, Juan Ramon Sanabria, Milena M. Santric Milicevic, Benn Sartorius, Sadaf G. Sepanlou, Masood Ali Shaikh, Mark G. Shrime, Vasiliki Stathopoulou, Muawiyyah Babale Sufiyan, Rizwan Suliankatchi Abdulkader, Rafael Tabarés-Seisdedos, Arash Tehrani-Banihashemi, Tesfalidet Tekelab, Mohamad-Hani Temsah, Bach Xuan Tran, Kingsley Nnanna Ukwaja, Olalekan A. Uthman, Vasiliy Victorovich Vlassov, Stein Emil Vollset, Tolassa Wakayo, Elisabete Weiderpass, Andrea Werdecker, Mohsen Yaghoubi, Mehdi Yaseri, Hassen Hamid Yimam, Naohiro Yonemoto, Maysaa El Sayed Zaki, Bassel Zein, Aisha O. Jumaan, Theo Vos, Simon I. Hay, Mohsen Naghavi, Christopher J. L. Murray, Ali H. Mokdad, Christina Fitzmaurice

**Affiliations:** Division of Hematology, Department of Medicine, Institute for Health Metrics and Evaluation, 2301 5th Avenue, Suite 600, UW Campus, Mailbox: 358210, Seattle, WA 98121 USA

**Keywords:** Eastern Mediterranean Region, Cancer, Mortality, Incidence, Disability-adjusted life years

## Abstract

**Objectives:**

To estimate incidence, mortality, and disability-adjusted life years (DALYs) caused by cancer in the Eastern Mediterranean Region (EMR) between 2005 and 2015.

**Methods:**

Vital registration system and cancer registry data from the EMR region were analyzed for 29 cancer groups in 22 EMR countries using the Global Burden of Disease Study 2015 methodology.

**Results:**

In 2015, cancer was responsible for 9.4% of all deaths and 5.1% of all DALYs. It accounted for 722,646 new cases, 379,093 deaths, and 11.7 million DALYs. Between 2005 and 2015, incident cases increased by 46%, deaths by 33%, and DALYs by 31%. The increase in cancer incidence was largely driven by population growth and population aging. Breast cancer, lung cancer, and leukemia were the most common cancers, while lung, breast, and stomach cancers caused most cancer deaths.

**Conclusions:**

Cancer is responsible for a substantial disease burden in the EMR, which is increasing. There is an urgent need to expand cancer prevention, screening, and awareness programs in EMR countries as well as to improve diagnosis, treatment, and palliative care services.

**Electronic supplementary material:**

The online version of this article (doi:10.1007/s00038-017-0999-9) contains supplementary material, which is available to authorized users.

## Introduction

With 8.7 million deaths (16% of all deaths), cancer was globally the second-leading cause of death behind cardiovascular diseases in 2015 (GBD 2015 Mortality and Causes of Death Collaborators 2016). There were 17.5 million incident cases globally, and cancer accounted for 209 million DALYs (GBD 2015 DALYs and HALE Collaborators 2016; GBD 2015 Disease and Injury Incidence and Prevalence Collaborators 2016; Global Burden of Disease Cancer Collaboration et al. 2016). In many countries, the epidemiological transition has led to a decrease in communicable, neonatal, maternal, and nutritional diseases, at the expense of an increase in non-communicable diseases over time (GBD 2015 DALYs and HALE Collaborators 2016). Prior studies examining cancer epidemiology in the EMR have either focused on a single year, a single country, or a particular component of cancer treatment (Aljurf et al. 2010; Abdel-Razeq et al. 2015; Kulhánová et al. 2017). What has not been analyzed for the EMR is how the epidemiological and demographical transition through an aging population, urbanization, industrialization, and lifestyle changes, as well political turmoil has affected the cancer burden (GBD 2015 DALYs and HALE Collaborators 2016). This evidence is essential for comprehensive cancer control planning. Given the diverse country profiles in the EMR with large differences in income, age structure, risk factor profile, and political stability, cancer prevention potential and treatment capacity requirements differ substantially between countries. In this study, we therefore present the Global Burden of Disease Study 2015 (GBD 2015) estimates of incidence, mortality, years of life lost (YLLs), years lived with disability (YLDs), and DALYs for 29 cancer groups and 22 EMR countries from 2005 to 2015 by age and sex, which to our knowledge is the most comprehensive assessment of cancer burden in the EMR (GBD 2015 DALYs and HALE Collaborators 2016; GBD 2015 Disease and Injury Incidence and Prevalence Collaborators 2016; GBD 2015 Mortality and Causes of Death Collaborators 2016; GBD 2015 Risk Factors Collaborators 2016). This quantitative assessment is especially important to guide health policy and to measure progress on the third Sustainable Development Goal (SDG) of reducing premature mortality from non-communicable diseases by one third by 2030 (United Nations 2016).

## Methods

The GBD 2015 study estimated incidence, prevalence, deaths, YLLs, YLDs, and DALYs for 195 countries and territories from 1990 to 2015. In total, 315 causes of diseases and injuries and 79 risk factors were systematically analyzed. Details of the methodology used in GBD 2015 to estimate general disease burden and cancer burden are described in detail elsewhere (GBD 2015 DALYs and HALE Collaborators 2016; GBD 2015 Disease and Injury Incidence and Prevalence Collaborators 2016; GBD 2015 Mortality and Causes of Death Collaborators 2016; GBD 2015 Risk Factors Collaborators 2016; Global Burden of Disease Cancer Collaboration et al. 2016).

Briefly, to estimate cancer burden, we mapped all neoplasms as defined by the 10th revision of the International Statistical Classification of Diseases (ICD-10) to one of the 29 GBD cancer groups. Input data for cancer mortality estimates came from vital registry mortality and cancer registry incidence data. The latter were transformed to mortality estimates using separately modeled mortality-to-incidence ratios (MIR) (Global Burden of Disease Cancer Collaboration et al. 2016). The raw data were processed to make them comparable and to account for “garbage codes”, which are codes assigned to causes that are not usable from a public health perspective (Naghavi et al. 2010). These causes were redistributed to the most likely underlying cause of death based on a regression model. Data were extracted at the most detailed level, by age group and sex, and mapped to the GBD cause list. Using a cause of death ensemble modeling (CODEm) approach with cause-specific covariates, we computed mortality estimates for each individual cause (Foreman et al. 2012). These estimates were scaled to fit into an independently modeled all-cause mortality estimate using the algorithm CodCorrect (GBD 2015 Mortality and Causes of Death Collaborators 2016). We transformed the final mortality estimates into incidence estimates using modeled MIR. Uncertainty from data sources and processing steps was propagated to the incidence estimates.

Cancer survival was calculated using a MIR-based scaling factor. We calculated 10-year prevalence of each cancer and each incidence cohort using these cancer survival estimates. The total prevalence was divided into four sequelae with variable disability weights: (1) diagnosis and treatment, (2) remission, (3) metastatic, and (4) terminal phase. We assumed a constant duration for sequelae (1), (3), and (4) for all countries over time. Duration of sequela (2) was the remaining prevalence after subtracting the duration of the fixed sequelae. We computed YLLs by multiplying deaths by the normative standard life expectancy at each age of death (GBD 2015 Mortality and Causes of Death Collaborators 2016). For each sequela, YLDs were calculated by multiplying the prevalence of each sequela by its disability weight. Finally, DALYs were calculated by summing YLLs and YLDs.

To analyze the contribution of population aging, population growth, and changes in age-specific incidence rates (ASIR) to the absolute change of cancer incidence, we calculated two scenarios. In the first, the age structure, sex structure, and age-specific rates from 2005 were applied to the 2015 population. The difference between the total number of cases in 2005 and the hypothetical scenario were attributed to population growth. In the second, the age-specific rates from 2005 were applied to the age structure, sex structure, and 2015 population. The differences between the two scenarios were attributed to population aging. Differences between the total number of cases in 2015 and the second hypothetical scenario were attributed to changes in the age-specific rates.

The 22 EMR countries were grouped according to per capita gross national income (GNI) into low-income countries (LICs) (Afghanistan, Djibouti, Somalia, and Yemen); middle-income countries (MICs) (Egypt, Iran, Iraq, Jordan, Lebanon, Libya, Morocco, Pakistan, Palestine, Sudan, Syria, and Tunisia); and high-income countries (HICs) (Bahrain, Kuwait, Oman, Qatar, Saudi Arabia, and the United Arab Emirates). LICs were defined as those having a per capita GNI of $1045 or less, MICs as those with a per capita GNI between $1046 and $12,735, and HICs as countries with per capita GNI of $12,736 or greater.

In this publication, all rates are reported per 100,000 person-years. We report 95% uncertainty intervals (UIs) for all estimates (listed in parentheses after the point estimates).

## Results

### Regional burden of cancer

Between 2005 and 2015 in the EMR region, incident cancer cases increased by 46.1% (34.5–59.4%) from 495 (457–537) thousand in 2005 to 723 (661–790) thousand cases in 2015 (Table 1). In 2015, cancer caused 379 (350–409) thousand deaths (Table 2) and 11.7 million (10.8–12.7 million) DALYs, of which 3% were attributable to YLDs and 97% to YLLs (eFig. 1). Age-standardized DALY rates (ASDR) remained unchanged between 2005 and 2015: 2663.7 (2486.3–2861.0) in 2005, and 2605.3 (2404.8–2816.0) in 2015 (eTable 4).

**Table 1:** Decomposition Analysis of Cancer Incidence by Country in the Eastern Mediterranean Region, both sexes, 2005–2015 (Global Burden of Disease Study 2015, Eastern Mediterranean Countries, 2005–2015)

Location	Number of incident cases		Expected number of cases in 2015		Change in incident cases 2005–2015 in %			Overall change in %
	2005	2015	Given population growth alone	Given population growth and aging	Due to population growth	Due to population ageing	Due to change in incidence rates	
Eastern Mediterranean Region	494,690	722,646	609,771	670,386	23.3	12.3	10.6	46.1
Afghanistan	25,015	36,809	33,400	35,375	33.5	7.9	5.7	47.2
Bahrain	663	1105	1050	1218	58.5	25.3	−17.1	66.7
Djibouti	772	1153	879	1020	13.8	18.3	17.3	49.4
Egypt	62,489	87,853	76,106	80,680	21.8	7.3	11.5	40.6
Iran	67,019	95,011	75,054	88,510	12.0	20.1	9.7	41.8
Iraq	28,593	41,208	38,474	40,354	34.6	6.6	3.0	44.1
Jordan	4016	6188	5677	6504	41.4	20.6	−7.9	54.1
Kuwait	1416	2544	2430	2701	71.5	19.2	−11.1	79.6
Lebanon	7446	13,272	10,867	11,933	45.9	14.3	18.0	78.2
Libya	5205	7646	5638	6762	8.3	21.6	17.0	46.9
Morocco	36,743	53,370	41,475	48,931	12.9	20.3	12.1	45.3
Oman	1127	2524	2016	2240	78.8	19.9	25.2	123.9
Pakistan	175,827	254,242	215,840	233,152	22.8	9.8	12.0	44.6
Palestine	2104	3479	2730	3053	29.8	15.3	20.3	65.4
Qatar	529	1290	1417	1281	167.7	−25.7	1.7	143.7
Saudi Arabia	9384	15,726	11,912	14,359	26.9	26.1	14.6	67.6
Somalia	7207	9862	9193	8974	27.6	−3.0	12.3	36.8
Sudan	20,552	29,740	25,871	27,940	25.9	10.1	8.8	44.7
Syria	7938	10,956	8134	9753	2.5	20.4	15.1	38.0
Tunisia	14,023	19,471	15,592	17,998	11.2	17.2	10.5	38.8
United Arab Emirates	3268	9247	6663	8585	103.9	58.8	20.2	182.9
Yemen	13,352	19,950	17,472	18,375	30.9	6.8	11.8	49.4

**Table 2:** Incidence, deaths and disability-adjusted life years for all cancers and 29 cancer groups in the Eastern Mediterranean Region, both sexes, 2015 (Global Burden of Disease Study 2015, Eastern Mediterranean Region, 2015)

Cause	Number of incident cases			Number of deaths			Number of DALYs (in thousands)		
	Males	Females	Both	Males	Females	Both	Males	Females	Both
All cancers groups	309,240 (282,640–340,657)	413,406 (361,086–467,300)	722,646 (660,722–790,102)	198,164 (181,894–217,561)	180,929 (160,360–202,560)	379,093 (350,252–408,580)	5865 (5354–6474)	5875 (5191–6608)	11,740 (10,800–12,742)
Lip and oral cavity cancer	14,068 (10,664–18,701)	15,358 (11,296–21,357)	29,426 (23,752–36,473)	4918 (4009–6069)	4837 (3841–5943)	9755 (8315–11,408)	161 (127–203)	151 (119–187)	312 (263–368)
Nasopharynx cancer	3219 (2080–4681)	1836 (1041–2849)	5055 (3606–6940)	1469 (1242–1776)	825 (680–984)	2294 (2006–2681)	52 (43–63)	30 (25–36)	82 (71–97)
Other pharynx cancer	4394 (3516–5394)	3593 (2915–4365)	7988 (6914–9273)	1654 (1410–1976)	1391 (1165–1641)	3045 (2679–3457)	46 (40–55)	40 (34–48)	87 (76–99)
Esophageal cancer	8795 (7449–10,517)	7992 (6474–9848)	16,788 (14,577–19,253)	9345 (8068–10,879)	8396 (6823–10,239)	17,741 (15,743–20,073)	265 (223–317)	251 (203–313)	516 (452–595)
Stomach cancer	27,093 (24,235–30,565)	17,725 (14,951–20,558)	44,818 (40,719–49,095)	17,462 (15,667–19,460)	11,847 (9954–13,631)	29,309 (26,728–31,947)	441 (394–496)	328 (264–387)	769 (690–851)
Colon and rectum cancer	18,662 (16,276–21,216)	17,150 (15,090–19,477)	35,813 (32,240–39,507)	13,268 (11,666–14,925)	13,148 (11,498–14,918)	26,416 (23,736–29,279)	392 (335–450)	373 (321–427)	764 (678–859)
Liver cancer	14,660 (12,042–16,784)	9908 (7418–11,788)	24,568 (20,618–27,385)	16,617 (13,869–18,735)	10,747 (8232–12,290)	27,365 (23,002–30,174)	448 (347–513)	292 (211–342)	740 (588–823)
Gallbladder and biliary tract cancer	2383 (1983–2803)	4543 (3731–5279)	6926 (5941–7838)	1985 (1673–2312)	3853 (3161–4465)	5839 (4973–6612)	50 (41–59)	98 (79–116)	148 (124–169)
Pancreatic cancer	6283 (5762–6843)	4885 (4393–5394)	11,168 (10,419–11,995)	7011 (6463–7643)	5480 (4917–6100)	12,491 (11,601–13,400)	179 (164–196)	129 (116–144)	308 (285–331)
Larynx cancer	11,975 (10,284–14,128)	2887 (2458–3493)	14,862 (13,083–17,018)	6477 (5676–7380)	1612 (1345–1917)	8090 (7222–9049)	172 (150–199)	46 (39–54)	218 (193–245)
Tracheal, bronchial and lung cancer	37,681 (32,768–42,292)	11,848 (10,591–13,321)	49,530 (44,083–54,564)	39,180 (34,316–43,815)	11,831 (10,442–13,380)	51,012 (45,430–56,191)	1013 (879–1144)	317 (277–364)	1330 (1171–1475)
Malignant skin melanoma	3021 (2072–3909)	2733 (2326–3212)	5755 (4863–6883)	617 (407–773)	514 (452–591)	1131 (947–1322)	20 (14–27)	16 (14–19)	36 (31–44)
Non-melanoma skin cancer	9359 (8443–10,285)	4697 (4165–5264)	14,056 (12,711–15,383)	1015 (921–1117)	314 (272–361)	1330 (1223–1449)	27 (24–30)	9 (7–10)	36 (33–39)
Breast cancer	2058 (1810–2359)	177,389 (148,702–207,371)	179,447 (150,924–209,304)	463 (408–530)	38,117 (32,305–44,251)	38,581 (32,795–44,698)	14 (12–16)	1314 (1101–1546)	1328 (1115–1561)
Cervical cancer	–	19,634 (14,721–25,505)	19,634 (14,721–25,505)	–	7878 (6158–9928)	7878 (6158–9928)	–	251 (192–323)	251 (192–323)
Uterine cancer	–	14,337 (11,576–17,621)	14,337 (11,576–17,621)	–	6857 (5641–8076)	6857 (5641–8076)	–	193 (157–228)	193 (157–228)
Ovarian cancer	–	10,946 (9024–13,395)	10,946 (9024–13,395)	–	6855 (5953–7833)	6855 (5953–7833)	–	235 (201–271)	235 (201–271)
Prostate cancer	27,533 (20,349–34,378)	–	27,533 (20,349–34,378)	13,861 (10,420–17,187)	–	13,861 (10,420–17,187)	243 (180–297)	–	243 (180–297)
Testicular cancer	3143 (2315–4266)	–	3143 (2315–4266)	1010 (792–1299)	–	1010 (792–1299)	52 (40–68)	–	52 (40–68)
Kidney cancer	5465 (4635–6345)	2856 (2463–3279)	8321 (7364–9305)	3497 (3046–3942)	1741 (1505–2039)	5239 (4699–5834)	110 (96–125)	60 (52–71)	170 (152–190)
Bladder cancer	23,449 (20,144–27,360)	6404 (5411–7716)	29,853 (26,404–33,966)	9452 (8524–10,545)	3151 (2723–3587)	12,604 (11,527–13,821)	217 (194–244)	73 (63–83)	289 (263–320)
Brain and nervous system	12,805 (9708–15,710)	11,045 (9432–12,702)	23,851 (20,099–27,075)	10,333 (7909–12,510)	8395 (7355–9338)	18,729 (16,185–20,983)	427 (321–525)	352 (300–392)	779 (666–881)
Thyroid cancer	3654 (2966–4357)	7536 (6026–9477)	11,191 (9589–13,565)	565 (484–688)	1112 (925–1352)	1678 (1478–2000)	17 (14–20)	32 (26–39)	49 (42–58)
Mesothelioma	839 (724–1015)	260 (215–339)	1099 (964–1313)	794 (717–909)	299 (245–360)	1093 (987–1243)	24 (22–28)	10 (8–12)	34 (30–39)
Hodgkin lymphoma	3247 (2539–4383)	2372 (1488–3502)	5619 (4436–7142)	1176 (932–1649)	811 (547–1245)	1987 (1655–2658)	50 (39–70)	36 (23–53)	86 (70–114)
Non-Hodgkin lymphoma	16,818 (14,017–20,617)	14,549 (9501–18,687)	31,367 (24,638–36,975)	6745 (5684–8121)	5999 (4194–7470)	12,744 (10,225–14,857)	251 (209–305)	216 (146–274)	466 (365–553)
Multiple myeloma	2693 (2323–3268)	2643 (2205–3157)	5336 (4694–6150)	2318 (2038–2761)	2395 (2028–2834)	4714 (4196–5411)	66 (57–80)	67 (56–80)	132 (116–154)
Leukemia	26,878 (23,330–31,115)	20,800 (17,703–24,679)	47,679 (42,513–53,365)	14,627 (13,388–16,013)	11,206 (10,045–12,467)	25,833 (24,105–27,809)	637 (579–705)	498 (445–557)	1135 (1053–1232)
Other neoplasms	19,054 (15,995–23,621)	17,468 (14,692–21,110)	36,523 (32,170–42,695)	12,292 (10,537–15,191)	11,306 (9694–13,334)	23,599 (20,803–27,877)	494 (418–603)	459 (389–546)	953 (840–1103)

### Regional age and sex variations in cancer burden

Females had higher ASIR in 2015 than males, with 199.6 (175.7–224.5) in females and 163.3 (150.2–178.7) in males (Table 3). Age-standardized mortality rate (ASMR) was higher in males compared to females at 113.8 (105.0–124.0) versus 95.8 (85.4–106.7), respectively. In females, breast cancer, leukemia, and cervical cancer were the most common incident cancers with 177 (149–207) thousand, 21 (18–25) thousand, and 20 (15–26) thousand cases, respectively (Table 2). The three cancers responsible for most cancer deaths in females were breast cancer with 38 (32–44) thousand deaths, colon and rectal cancer with 13 (11–15) thousand deaths, and stomach cancer with 12 (10–14) thousand deaths. The top three causes of DALYs in females were breast cancer with 1.3 (1.1–1.5) million DALYs, leukemia with 498 (445–557) thousand DALYs, and other neoplasms with 459 (339–546) thousand DALYs (Table 2).

**Table 3:** Age-standardized incidence, mortality and DALY rates per 100,000 for the Eastern Mediterranean Region and its 22 countries, both sexes, 2015 (Global Burden of Disease Study 2015, Eastern Mediterranean Countries, 2015)

Location	Age-standardized incidence rate			Age-standardized mortality rate			Age-standardized DALY rate		
	Males	Females	Both	Males	Females	Both	Males	Females	Both
Eastern Mediterranean Region	163 (150–179)	200 (176–225)	180 (166–195)	114 (105–124)	96 (85–107)	104 (97–112)	2651 (2435–2915)	2583 (2287–2897)	2605 (2404–2816)
Afghanistan	178 (127–237)	345 (171–642)	259 (161–411)	156 (109–205)	177 (102–276)	165 (112–222)	3546 (2355–4889)	5066 (2618–8449)	4260 (2634–6153)
Bahrain	134 (105–168)	156 (122–196)	138 (117–162)	88 (69–107)	70 (56–85)	77 (66–89)	1752 (1385–2170)	1776 (1395–2221)	1710 (1453–2003)
Djibouti	201 (103–409)	221 (105–528)	210 (113–388)	158 (80–311)	129 (61–301)	142 (75–257)	3972 (1896–8438)	3634 (1696–8796)	3783 (1951–7187)
Egypt	146 (135–165)	137 (126–149)	139 (131–151)	100 (94–111)	65 (62–71)	81 (77–87)	2437 (2299–2619)	1846 (1740–1973)	2113 (2022–2229)
Iran	208 (166–260)	135 (104–175)	173 (146–205)	123 (98–150)	69 (52–89)	97 (81–113)	2650 (2080–3338)	1704 (1286–2243)	2190 (1825–2595)
Iraq	185 (129–243)	254 (178–344)	220 (172–280)	138 (95–176)	117 (86–156)	126 (101–155)	3340 (2303–4462)	3329 (2385–4498)	3318 (2625–4180)
Jordan	153 (131–179)	145 (115–178)	147 (129–169)	98 (84–114)	64 (53–76)	80 (71–90)	2193 (1888–2580)	1708 (1423–2039)	1939 (1713–2179)
Kuwait	139 (118–167)	179 (148–214)	156 (139–176)	71 (60–84)	68 (56–82)	70 (62–79)	1403 (1181–1664)	1523 (1256–1815)	1453 (1292–1644)
Lebanon	284 (192–391)	246 (165–341)	262 (195–336)	158 (104–218)	110 (73–146)	133 (99–170)	3302 (2179–4603)	2715 (1771–3746)	2995 (2238–3864)
Libya	226 (187–276)	160 (133–194)	189 (166–218)	157 (128–191)	91 (74–110)	121 (104–139)	3326 (2703–4039)	2245 (1815–2752)	2752 (2360–3181)
Morocco	180 (126–258)	210 (138–296)	195 (151–252)	140 (98–201)	108 (72–148)	122 (96–159)	3019 (2053–4385)	2690 (1776–3789)	2844 (2205–3728)
Oman	121 (97–143)	118 (98–144)	116 (99–132)	74 (58–87)	58 (47–69)	66 (55–75)	1564 (1237–1867)	1397 (1115–1711)	1469 (1220–1688)
Pakistan	153 (127–185)	278 (213–350)	214 (180–253)	109 (91–131)	126 (97–155)	117 (100–135)	2878 (2374–3537)	3498 (2678–4319)	3182 (2713–3685)
Palestine	149 (115–192)	153 (117–205)	150 (122–182)	116 (87–146)	71 (55–92)	92 (74–110)	2816 (2094–3653)	2015 (1546–2663)	2396 (1925–2898)
Qatar	144 (110–189)	180 (136–226)	150 (122–183)	90 (67–118)	79 (59–99)	84 (67–104)	1667 (1251–2170)	1932 (1452–2463)	1700 (1382–2109)
Saudi Arabia	104 (85–128)	92 (73–113)	96 (82–111)	67 (60–77)	43 (39–49)	55 (50–60)	1300 (1154–1490)	992 (881–1124)	1134 (1033–1251)
Somalia	144 (62–304)	257 (76–608)	202 (69–449)	130 (53–270)	166 (44–382)	149 (49–319)	3267 (1266–7474)	4751 (1262–11,703)	4031 (1274–9504)
Sudan	136 (97–185)	163 (97–241)	149 (110–195)	111 (80–157)	85 (54–122)	97 (73–125)	2485 (1719–3601)	2216 (1338–3228)	2338 (1722–3074)
Syria	103 (84–124)	105 (86–126)	103 (90–118)	76 (61–92)	54 (44–63)	64 (55–72)	1653 (1343–1972)	1345 (1099–1592)	1487 (1288–1685)
Tunisia	224 (169–289)	164 (117–213)	190 (151–231)	163 (122–210)	76 (55–100)	115 (90–141)	3445 (2596–4545)	1878 (1356–2479)	2610 (2057–3170)
United Arab Emirates	213 (155–290)	226 (158–310)	207 (157–270)	106 (81–139)	85 (65–115)	97 (76–123)	2191 (1621–2891)	2279 (1694–3069)	2145 (1651–2720)
Yemen	131 (85–198)	201 (114–353)	167 (101–268)	109 (72–168)	103 (59–164)	106 (65–164)	2445 (1529–3909)	2753 (1558–4552)	2601 (1527–4180)

The most common incident cancers in males in 2015 were tracheal, bronchus, and lung cancer (TBL) with 38 (33–42) thousand cases, followed by prostate cancer and stomach cancer, with 28 (20–34) thousand and 27 (24–31) thousand cases, respectively. These cancers accounted for 30% of the incidence of all cancers. The most common causes of cancer deaths in males were TBL, stomach cancer, and liver cancer with 39 (34–44) thousand, 17 (16–19) thousand, and 17 (14–19) thousand deaths, respectively. The top three causes of DALYs in males were TBL with 1.0 (0.8–1.1) million DALYs, leukemia with 637 (579–705) thousand DALYs, and other neoplasms with 494 (418–603) thousand DALYs (Table 2).

**Fig. 1:**
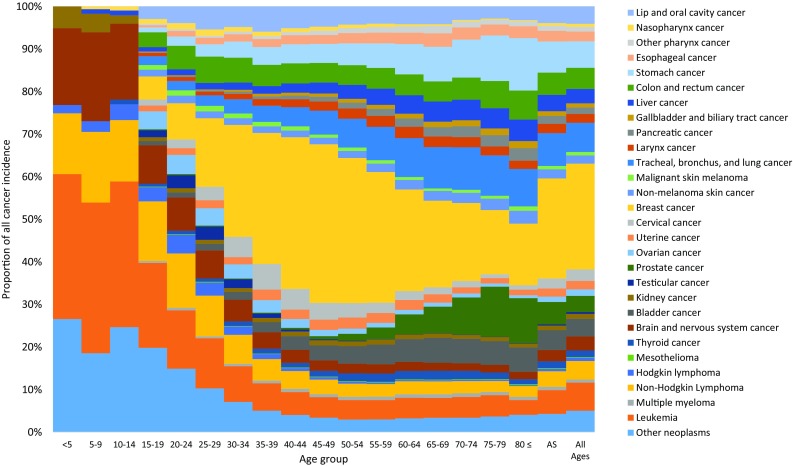
Age-specific contribution of cancer groups to total cancer incidence in the Eastern Mediterranean Region, both sexes, 2015 (Global Burden of Disease Study 2015, Eastern Mediterranean Region, 2015)

**Fig. 2:**
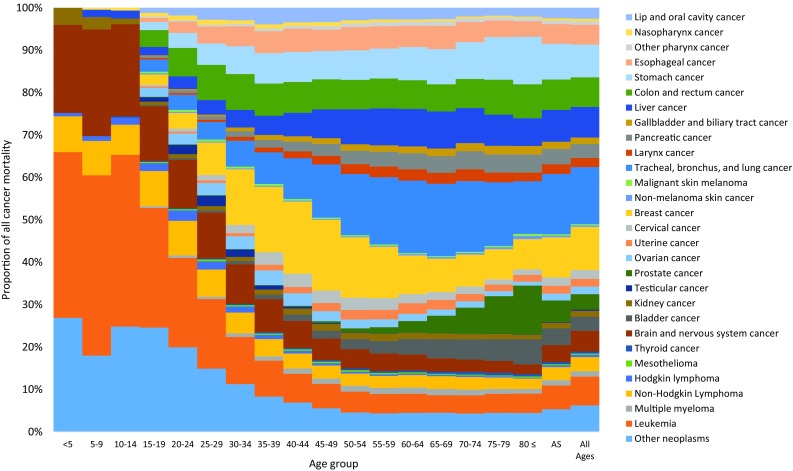
Age-specific contribution of cancer groups to total cancer mortality in the Eastern Mediterranean Region, both sexes, 2015. (Global Burden of Disease Study 2015, Eastern Mediterranean Region, 2015)

In children aged 0–14 years, the most common cancers were leukemia, other neoplasms, and cancer of the brain and nervous system (Fig. 1). These cancers were also the ones responsible for most childhood cancer deaths (Fig. 2). In adolescents and young adults (ages 15–39 years), the most common cancers were breast cancer, followed by leukemia and other neoplasms. These cancers were also main causes of death in this age group.

### National cancer incidence, mortality, and burden

In 2015, Lebanon had the highest ASIR for all cancers at 261.9 (194.6–336.2), followed by Afghanistan at 258.8 (161.3–411.3), and Iraq at 219.9 (172.2–279.8) (Table 3). ASMRs were highest in Afghanistan at 165.0 (111.5–221.7), followed by Somalia at 148.6 (49.1–319.1), and Djibouti at 142.0 (75.4–256.6). Those three countries also had the highest ASDRs in 2015. Saudi Arabia, Syria, and Oman had the lowest ASIRs in 2015 with 95.6 (82.3–111.5), 103.5 (89.9–117.8), and 115.8 (98.5–131.8), respectively. Those countries also had the lowest ASMR with 54.8 (50.1–60.0), 64.0 (55.0–72.4), and 66.0 (55.2–74.7), respectively. Along with Kuwait, these countries had the lowest ASDRs in 2015 as well.

### Burden of different cancer groups

Excluding the “other neoplasms” group, five cancers, namely breast cancer, TBL, leukemia, stomach cancer, and colon and rectal cancer ranked highest in terms of incident cases in the region. Breast cancer had the highest ASIR in the EMR in 2015 with 42.3 (35.7–48.9) cases. It also had the second-highest ASMR after TBL in the region with 9.9 (8.5–11.3) deaths (eTable 1). There were 179 (151–209) thousand new cases in 2015, 39 (33–45) thousand deaths, and 1.3 (1.1–1.6) million DALYs caused by breast cancer (Table 2). Only 1% (2058 cases) of breast cancer cases occurred in males (Table 2). Nine percent of all DALYs caused by breast cancer came from YLDs (eFigure 1).

In 2015, TBL had the second highest ASIR the region with 13.9 (12.5–15.2). It was the leading cause of cancer deaths and DALYs in the region with 51 (44–55) thousand incident cases, 51 (45–56) thousand deaths, and 1.3 (1.2–1.5) million DALYs. Seventy-six percent of new cases and deaths occurred in males. Only 1% of DALYs came from YLDs.

There were 48 (43–53) thousand new cases of leukemia in 2015 in the region and 26 (24–28) thousand deaths, making it the third most common cancer in the region. Leukemia caused 1.1 (1.1–1.2) million DALYs, with 97% coming from YLLs.

Stomach cancer had the fourth-highest ASIR the region in 2015 at 13.2 (12.0–14.4), but ranked first in Afghanistan with 40.5 (26.1–55.0) and second in Iran, Yemen, and Sudan with 29.1 (24.0–35.7), 19.0 (11.6–29.4), and 18.1 (13.2–23.6), respectively (Online Appendix Data). There were 45 (41–49) thousand cases in 2015, 29 (27–32) thousand deaths, and 769 (690–851) thousand DALYs, of which only 2% came from YLDs. Sixty percent of incident cases, 60% of deaths, and 57% of DALYs occurred in males.

Colon and rectum cancer was the sixth most frequent cancer in the region in 2015 with 36 (32–40) thousand incident cases, 26 (24–29) thousand deaths, and 764 (678–859) thousand DALYs. It was the second most frequent incident cancer in 2015 in Jordan, Kuwait, Lebanon, Libya, Qatar, and Saudi Arabia.

### Drivers of change in cancer incidence

Between 2005 and 2015, the overall change in the number of incident cancer cases ranged between 36.8% in Somalia and 182.9% in the UAE (Table 1). High-income EMR countries in addition to Lebanon experienced the largest increase in cancer incidence, which was mainly driven by population growth in all countries. Population aging was responsible for 12% of the increase in incident cancer cases in the region in total, ranging from −25.7% in Qatar to 58.8% in the UAE. Change in age-specific incident rates ranged between −17.1% in Bahrain and 25.2% in Oman relative to the overall change in incident cases (Table 1).

## Discussion

In 2015, cancer was responsible for 9.4% (8.9–9.9%) of all deaths and 5.1% (4.6–5.8%) of all DALYs in the EMR countries compared to 15.7% (15.5–15.9%) of deaths and 8.5% (7.8–9.2%) of all DALYs at the global level (GBD 2015 DALYs and HALE Collaborators 2016; GBD 2015 Mortality and Causes of Death Collaborators 2016). This puts cancer as the third-leading cause of death and the eighth-leading cause of DALYs in the EMR. In EMR countries, cancer deaths between 2005 and 2015 have increased by 32.9%. Females experienced higher cancer incidence in the EMR but lower cancer deaths compared to males, which can be explained by less aggressive cancers (breast, cervical) being among the top cancers in females compared to males (lung, stomach). Age-standardized cancer incidence varied substantially between EMR countries with infection-related cancers playing a more important role in low- and low-middle income countries (e.g., stomach cancer having the highest ASIR in Afghanistan, Iran, Yemen, and Sudan, and cancers related to low physical activity and cancers with strong lifestyle-related risk factors such as colorectal cancer being more common in middle- and high-income EMR countries such as Lebanon, the UAE, and Libya).

Given this alarming trend and the substantial contribution of cancer to the disease burden in EMR countries, cancer control has to be among the top health policy priorities. Compared to other studies (Aljurf et al. 2010; Abdel-Razeq et al. 2015; Kulhánová et al. 2017) analyzing cancer burden in the EMR countries, the GBD study provides analyses of all diseases over time, which means that cancer can be viewed in the context of other health priorities. Kulhánová et al. recently published an analysis using GLOBOCAN data to analyze the cancer burden in the EMR (Kulhánová et al. 2017). Because of different methods to estimate incidence and mortality as well as few high-quality data sources for cancer incidence and mortality in the EMR, GBD estimates for incidence differ between 50% fewer incident cases (for Syria) to 215% more incident cases (for the UAE). For mortality, GBD estimates range from 56% fewer deaths in Syria to 153% more deaths in the UAE (eTable 4). Whereas the GLOBOCAN methodology starts with estimating cancer incidence and then for most EMR countries models survival to estimate mortality (Ferlay et al. 2015), GBD uses cancer registry incidence-based mortality estimates as well as vital registration data to model mortality and then uses these mortality estimates as well as modeled MIR to estimate cancer incidence. An advantage of the GBD study is the ability to compare trends over time, which allows for analysis of the effects of the demographical and epidemiological transition, and also the effectiveness of public health policies. The discrepancies between GLOBOCAN and GBD estimates underscore the need for better data to assess cancer burden in the EMR countries. Few high-quality population-based cancer registries exist in the EMR, with the Global Initiative for Cancer Registry Development (GICR) actively promoting further development of cancer registries (International Agency for Research on Cancer (IARC) 2011). At the same time, strengthening of vital registration systems and integration of surveillance systems for other non-communicable diseases is needed in the region. Until better data become available, model-based estimates have to be used to guide local policy.

Two significant international statements address the threat of non-communicable diseases and propose interventions as well as metrics to measure success. The SDGs, as the successors of the Millennium Development Goals, which shaped public health policy for 15 years, now include non-communicable diseases (NCDs) in the third goal, “by 2030, reduce by one-third premature mortality from non-communicable diseases through prevention and treatment and promote mental health and well-being” (United Nations 2016). Control of NCDs has also been targeted in the WHO Global Action Plan for Prevention and Control of NCDs 2013–2020 (World Health Organization 2013). Our study shows that substantial efforts are required in most EMR countries to meet the SDG targets of reducing cancer mortality. Culprits for the disappointing pace of cancer control to date can be found in all aspects of cancer care, from primary prevention and screening, to early diagnosis, access to cancer treatment, tertiary prevention, and palliative care (World Health Organization 2014).

We have seen exciting advances in our understanding of cancer and resulting treatment approaches in the last decade. However, the increasing cancer burden due to an aging population and the exploding costs associated with complex cancer treatments are leading to unacceptable increases in health care expenditure, which will be impossible to sustain for most countries (Kelly and Smith 2014). For this reason, risk factor reduction has to be a priority for any cancer control effort. The top five risk factors identified in GBD as contributing to cancer mortality in the EMR are tobacco, dietary risks, high body mass index, occupational risks, and air pollution (GBD 2015 Risk Factors Collaborators 2016). With lung cancer being the second-leading cause of cancer death in the region, tobacco control has to be the top priority. Health hazards of cigarette smoking are well established. However, other forms of tobacco consumption such as chewing and shisha (waterpipe) smoking also lead to an increased risk of death, mainly due to cancer (Etemadi et al. 2016). All countries in the EMR with the exception of Somalia and Palestine have signed the WHO Framework Convention on Tobacco Control (WHO FCTC), and all countries except Morocco have ratified it. However, in many EMR countries, smoking rates have not declined, with certain forms of tobacco consumption such as shisha smoking even rising (Maziak et al. 2015). Secondhand smoking is not restricted in most countries in the EMR despite the FCTC recommendations (Heydari et al. 2012).

Obesity and lack of physical activity are risk factors that also follow a dangerous trend, with obesity prevalence rising in many EMR countries to concerning levels (Ng et al. 2014). Obesity has been proven to be a risk factor for esophageal adenocarcinoma, colon, rectal, kidney, and pancreas cancer, gallbladder cancer in females, and postmenopausal breast, ovarian, and uterine cancers (Lauby-Secretan et al. 2016). Physical inactivity has been linked to an increased risk for cancer, especially colon and breast cancer (American Institute for Cancer Research and World Cancer Research Fund 2007). For both sexes combined, breast cancer is the most common incident cancer in every EMR country, and colorectal cancer is among the top four most common cancers in all high-income EMR countries as well as all middle-income EMR countries except for Iran, Pakistan, Sudan, Syria, Egypt, Morocco, and Iraq. This stresses the importance of health intervention programs and environmental policies to increase physical activity and healthy dietary habits.

Other important strategies for primary prevention include vaccination against human papillomavirus (HPV) for cervical cancer prevention, as well as hepatitis B vaccination and treatment of hepatitis B and C, especially in countries with high hepatitis C prevalence such as Egypt, where liver cancer is the leading cause of cancer death (Alavian and Haghbin 2016). In the case of liver cancer, screening of high-risk groups has also been recommended by the National Comprehensive Cancer Network (NCCN) as a core intervention in the resources stratified guidelines (National Comprehensive Cancer Network 2016). However, early detection is dependent on a functioning primary care system as well as universal access to care, the developments of both of which are hampered by fragmented care systems, lack of strategic planning, an unregulated private sector, as well as political turmoil in some EMR countries (Regional Committee for the EM/RC57/Tech.Disc.1 and Eastern Mediterranean 2010).

An emphasis on addressing cancer once it becomes clinically symptomatic rather than on detecting it early is also apparent by the lack of population-wide cancer screening programs. Effective screening is currently available for cervical cancer, colorectal cancer, breast cancer, oral cancer, and stomach cancer (in high-risk populations) (Sankaranarayanan 2014). With breast and cervical cancer being among the most common cancers in females in every EMR country, cancer screening should be among the prioritized prevention efforts (Goldie et al. 2005; Yip et al. 2008). For screening programs to be successful at the population level, strategic implementation should be coordinated at the national level and include educational components, as well monitoring and evaluation to ensure success and sustainability. Opportunistic screening programs in the past have been hampered by low participation rates due to anxiety, misperception of the screening’s purpose, and a general sense that cancer at any stage is a death sentence (Al Mulhim et al. 2015; Al-Zalabani et al. 2016). Civil engagement through education and advocacy is therefore an important pillar of successful and sustainable screening programs.

Cancer treatment programs depend on multidisciplinary approaches that are often unavailable in EMR countries. Laboratory services, pathology, radiology, oncology nursing, surgery, medical and radiation oncology, pharmacology, transfusion services, nutritional and psychosocial support services, as well as palliative care and hospice services are the core disciplines required to provide cancer care. There is a lack of human capital for many, if not of all, of these disciplines, which will require continued and coordinated efforts to train local staff and ensure retention (World Health Organization 2014).

Another important factor contributing to poor health in some EMR countries is war, leading to a large number of displaced people, disruption in care structures and supplies, lack of qualified healthcare personnel, and financial strains on patients and healthcare systems in countries with large refugee populations (Spiegel et al. 2014; Sahloul et al. 2016). Innovative solutions to monitor disease burden in these most vulnerable populations have been proposed and include web-based cancer registries with linkages between countries (Spiegel et al. 2014). Approaches during humanitarian emergencies to prevent and treat cancer and other diseases requiring complex care systems include clear referral guidelines as provided by the United Nations High Commissioner for Refugees (UNHCR), as well as financing systems such as health insurance or social security (United Nations High Commissioner for Refugees 2009, 2012).

### Limitations

Our data sources to estimate cancer burden in the EMR are vital registration and cancer registry data. The GBD study tries to identify and utilize all available data sources in the estimation process. However, data sources in low- and middle-income countries are scarce, and causes of death and cancer registration is not a routine procedure in many health care systems. Even when civil registration exits, war or civil unrest can interrupt routine data collection. In the absence of reliable data, our estimates are largely driven by regional trends and the selection of model covariates which lead consequently to wider uncertainty intervals and time trends that are therefore often non-significant. Furthermore, miscoding of causes of death—as the so-called garbage codes—in vital registration data can influence both our mortality estimates and incidence estimates. Misclassifying metastatic sites (e.g., lung, liver, bone) as the primary cancer site or as second cancers is another potential source of bias. This is particularly true in countries with low-quality registration and limited diagnostic sources.

### Conclusions

Cancer is among the leading causes of death and DALYs in most EMR countries. Prioritization of different aspects of the cancer control continuum depends on local health infrastructure as well as disease epidemiology. Given the dramatic increase in cancer cases and deaths over the last decade, all stakeholders including health policymakers, care providers, and the general public need to actively engage to define these priorities and work together on implementation of evidence-based cancer control strategies.

## Electronic supplementary material

Below is the link to the electronic supplementary material.

Supplementary material 1 (XLSX 3042 kb)

Supplementary material 2 (XLSX 27 kb)

Supplementary material 3 (DOCX 78 kb)
