# Supplementary material for: Burden of cancer in the Eastern Mediterranean Region, 2005–2015: findings from the Global Burden of Disease 2015 Study
Source: Int J Public Health. 2017 Aug 3;63(Suppl 1):151–64. doi: 10.1007/s00038-017-0999-9 (PMC5973975; doi:10.1007/s00038-017-0999-9)
Supplement: Supplementary file 3 — Supplementary material 3 (DOCX 78 kb) [file 38_2017_999_MOESM3_ESM.docx]

Electronic Supplementary Material

**Article title:**

Burden of cancer in the Eastern Mediterranean Region, 2005–2015: Findings from the Global Burden of Disease 2015 study

**Journal:**

International Journal of Public Health

**Authors:**

GBD 2015 Eastern Mediterranean Region Cancer Collaborators

**Corresponding author:**

Christina Fitzmaurice, MD, MPH

Assistant Professor

Department of Medicine, Division of Hematology

Institute for Health Metrics and Evaluation

2301 5th Avenue, Suite 600, Seattle, WA 98121, USA

UW Campus Mailbox: 358210

cf11@uw.edu | http://healthdata.org

| eTable 1. Age-standardized Incidence, Mortality and DALY Rates per 100.000 for 29 Cancer Groups in the Eastern Mediterranean Region, both sexes, 2015. DALY = disability-adjusted life-years. (Global Burden of Disease Study 2015, Eastern Mediterranean Region, 2015) | | | | | | | | | |
| --- | --- | --- | --- | --- | --- | --- | --- | --- | --- |
| Cause | Age-standardized Incidence Rate | | | Age-standardized Mortality Rate | | | Age-standardized DALY Rate | | |
|  | Males | Females | Both | Males | Females | Both | Males | Females | Both |
| All Cancer Groups | 163 (150 - 179) | 200 (176 - 225) | 180 (166 - 195) | 114 (105 - 124) | 96 (85 - 107) | 104 (97 - 112) | 2651 (2435 - 2915) | 2583 (2287 - 2897) | 2605 (2404 - 2816) |
| Lip and oral cavity cancer | 7 (5 - 8) | 7 (6 - 10) | 7 (6 - 9) | 3 (2 - 3) | 3 (2 - 3) | 3 (2 - 3) | 69 (56 - 85) | 69 (55 - 85) | 69 (59 - 81) |
| Nasopharynx cancer | 1 (1 - 2) | 1 (0 - 1) | 1 (1 - 1) | 1 (1 - 1) | 0 (0 - 0) | 1 (0 - 1) | 21 (18 - 25) | 12 (10 - 15) | 17 (15 - 20) |
| Other pharynx cancer | 2 (2 - 3) | 2 (2 - 2) | 2 (2 - 3) | 1 (1 - 1) | 1 (1 - 1) | 1 (1 - 1) | 23 (19 - 27) | 19 (16 - 23) | 21 (19 - 24) |
| Esophageal cancer | 5 (4 - 6) | 4 (3 - 5) | 5 (4 - 5) | 5 (5 - 6) | 4 (4 - 5) | 5 (4 - 5) | 123 (106 - 144) | 116 (94 - 143) | 119 (105 - 135) |
| Stomach cancer | 17 (15 - 19) | 10 (9 - 11) | 13 (12 - 14) | 11 (10 - 12) | 7 (6 - 8) | 9 (8 - 9) | 223 (201 - 250) | 156 (128 - 181) | 189 (171 - 207) |
| Colon and rectum cancer | 10 (9 - 11) | 9 (8 - 10) | 9 (9 - 10) | 7 (7 - 8) | 7 (7 - 8) | 7 (7 - 8) | 176 (154 - 199) | 175 (152 - 199) | 175 (157 - 195) |
| Liver cancer | 8 (7 - 9) | 6 (4 - 7) | 7 (6 - 8) | 10 (8 - 11) | 6 (5 - 7) | 8 (7 - 9) | 218 (177 - 246) | 142 (106 - 164) | 180 (148 - 199) |
| Gallbladder & biliary tract cancer | 1 (1 - 2) | 3 (2 - 3) | 2 (2 - 2) | 1 (1 - 1) | 2 (2 - 3) | 2 (2 - 2) | 25 (21 - 30) | 50 (40 - 58) | 37 (32 - 42) |
| Pancreatic cancer | 4 (3 - 4) | 3 (3 - 3) | 3 (3 - 4) | 4 (4 - 5) | 3 (3 - 4) | 4 (4 - 4) | 90 (83 - 98) | 68 (61 - 76) | 79 (73 - 85) |
| Larynx cancer | 6 (6 - 7) | 1 (1 - 2) | 4 (3 - 4) | 4 (3 - 4) | 1 (1 - 1) | 2 (2 - 3) | 87 (76 - 99) | 22 (19 - 26) | 54 (49 - 61) |
| Tracheal, Bronchial and Lung Cancer | 21 (19 - 24) | 7 (6 - 7) | 14 (12 - 15) | 23 (20 - 26) | 7 (6 - 8) | 15 (13 - 16) | 509 (444 - 570) | 155 (136 - 177) | 333 (295 - 368) |
| Malignant skin melanoma | 1 (1 - 2) | 1 (1 - 2) | 1 (1 - 2) | 0 (0 - 0) | 0 (0 - 0) | 0 (0 - 0) | 9 (6 - 11) | 7 (6 - 8) | 8 (7 - 10) |
| Non-melanoma skin cancer | 5 (5 - 6) | 3 (2 - 3) | 4 (4 - 4) | 1 (1 - 1) | 0 (0 - 0) | 0 (0 - 0) | 14 (13 - 15) | 4 (4 - 5) | 9 (8 - 10) |
| Breast cancer | 1 (1 - 1) | 85 (71 - 98) | 42 (36 - 49) | 0 (0 - 0) | 20 (17 - 23) | 10 (8 - 11) | 7 (6 - 8) | 589 (501 - 685) | 291 (248 - 338) |
| Cervical cancer | - | 9 (7 - 11) | 4 (3 - 5) | - | 4 (3 - 5) | 2 (2 - 3) | - | 114 (88 - 145) | 56 (43 - 71) |
| Uterine cancer | - | 7 (6 - 8) | 3 (3 - 4) | - | 4 (3 - 4) | 2 (2 - 2) | - | 95 (78 - 112) | 47 (39 - 55) |
| Ovarian cancer | - | 5 (4 - 5) | 2 (2 - 3) | - | 3 (3 - 4) | 2 (1 - 2) | - | 100 (86 - 114) | 49 (42 - 56) |
| Prostate cancer | 20 (14 - 24) | - | 9 (7 - 12) | 11 (8 - 14) | - | 5 (4 - 7) | 166 (125 - 204) | - | 81 (61 - 99) |
| Testicular cancer | 1 (1 - 1) | - | 1 (0 - 1) | 0 (0 - 0) | - | 0 (0 - 0) | 15 (12 - 20) | - | 8 (6 - 10) |
| Kidney cancer | 3 (2 - 3) | 1 (1 - 1) | 2 (2 - 2) | 2 (2 - 2) | 1 (1 - 1) | 1 (1 - 2) | 48 (42 - 54) | 25 (22 - 29) | 37 (33 - 41) |
| Bladder cancer | 13 (12 - 15) | 4 (3 - 4) | 8 (7 - 9) | 6 (6 - 7) | 2 (2 - 2) | 4 (4 - 5) | 121 (109 - 135) | 39 (34 - 45) | 80 (73 - 88) |
| Brain and nervous system | 5 (4 - 6) | 4 (4 - 5) | 5 (4 - 5) | 5 (4 - 5) | 4 (3 - 4) | 4 (4 - 5) | 150 (115 - 183) | 128 (111 - 142) | 139 (120 - 156) |
| Thyroid cancer | 2 (2 - 2) | 4 (3 - 5) | 3 (2 - 4) | 0 (0 - 0) | 1 (1 - 1) | 1 (0 - 1) | 8 (7 - 10) | 16 (13 - 20) | 12 (11 - 15) |
| Mesothelioma | 0 (0 - 1) | 0 (0 - 0) | 0 (0 - 0) | 0 (0 - 0) | 0 (0 - 0) | 0 (0 - 0) | 11 (10 - 12) | 4 (3 - 5) | 7 (7 - 8) |
| Hodgkin lymphoma | 1 (1 - 2) | 1 (1 - 1) | 1 (1 - 1) | 1 (0 - 1) | 0 (0 - 1) | 0 (0 - 1) | 17 (14 - 24) | 13 (8 - 19) | 15 (13 - 20) |
| Non-Hodgkin lymphoma | 7 (6 - 8) | 6 (4 - 8) | 7 (5 - 8) | 3 (3 - 4) | 3 (2 - 4) | 3 (3 - 4) | 96 (81 - 116) | 87 (60 - 109) | 92 (73 - 108) |
| Multiple myeloma | 1 (1 - 2) | 1 (1 - 2) | 1 (1 - 2) | 1 (1 - 2) | 1 (1 - 2) | 1 (1 - 2) | 31 (27 - 37) | 33 (27 - 39) | 32 (28 - 37) |
| Leukemia | 11 (10 - 13) | 9 (8 - 10) | 10 (9 - 11) | 7 (6 - 7) | 5 (5 - 6) | 6 (5 - 6) | 218 (199 - 238) | 175 (157 - 194) | 196 (184 - 212) |
| Other neoplasms | 8 (7 - 10) | 7 (6 - 9) | 8 (7 - 9) | 6 (5 - 8) | 5 (5 - 6) | 6 (5 - 7) | 176 (151 - 215) | 169 (145 - 200) | 173 (152 - 201) |

| eTable 2. Incidence, Deaths and Disability-Adjusted Life Years for the Eastern Mediterranean Region and its 22 countries, both sexes, 2015. (Global Burden of Disease Study 2015, Eastern Mediterranean Countries, 2015) | | | | | | | | | |
| --- | --- | --- | --- | --- | --- | --- | --- | --- | --- |
| Location | Number of Incident Cases | | | Number of Deaths | | | Number of DALYs (in thousands) | | |
|  | Males | Females | Both | Males | Females | Both | Males | Females | Both |
| Eastern Mediterranean Region | 309240 (282640 - 340657) | 413406 (361086 - 467300) | 722646 (660722 - 790102) | 198164 (181894 - 217561) | 180929 (160360 - 202560) | 379093 (350252 - 408580) | 5865 (5354 - 6474) | 5875 (5191 - 6608) | 11740 (10800 - 12742) |
| Afghanistan | 10710 (7366 - 14838) | 26099 (11609 - 50796) | 36809 (20719 - 62375) | 8303 (5476 - 11581) | 11657 (6065 - 19343) | 19960 (12339 - 28796) | 264 (169 - 385) | 419 (207 - 724) | 683 (403 - 1024) |
| Bahrain | 527 (408 - 668) | 577 (443 - 744) | 1105 (924 - 1326) | 272 (212 - 344) | 204 (160 - 259) | 476 (399 - 565) | 9 (7 - 11) | 7 (6 - 9) | 16 (13 - 19) |
| Djibouti | 508 (245 - 1098) | 645 (297 - 1596) | 1153 (600 - 2213) | 368 (173 - 780) | 347 (159 - 849) | 715 (363 - 1364) | 12 (6 - 26) | 12 (5 - 28) | 24 (12 - 45) |
| Egypt | 41479 (38278 - 46189) | 46374 (42594 - 50260) | 87853 (82803 - 94912) | 26520 (25027 - 28630) | 20874 (19754 - 22406) | 47394 (45414 - 50343) | 796 (751 - 850) | 671 (631 - 714) | 1467 (1403 - 1539) |
| Iran | 55327 (43643 - 69852) | 39684 (29717 - 52991) | 95011 (79386 - 113095) | 31086 (24386 - 38893) | 18093 (13313 - 23728) | 49179 (40814 - 58144) | 793 (616 - 1028) | 544 (409 - 732) | 1338 (1105 - 1602) |
| Iraq | 15413 (10790 - 20733) | 25795 (17620 - 35932) | 41208 (31452 - 53209) | 10065 (6933 - 13432) | 10728 (7648 - 14509) | 20793 (16441 - 26207) | 341 (239 - 460) | 379 (270 - 516) | 720 (562 - 915) |
| Jordan | 2984 (2575 - 3473) | 3204 (2562 - 3940) | 6188 (5407 - 7089) | 1705 (1462 - 2011) | 1273 (1060 - 1527) | 2978 (2633 - 3342) | 51 (44 - 59) | 43 (36 - 51) | 94 (83 - 105) |
| Kuwait | 1175 (984 - 1432) | 1369 (1101 - 1683) | 2544 (2236 - 2935) | 504 (417 - 607) | 418 (342 - 508) | 922 (814 - 1059) | 16 (13 - 19) | 14 (12 - 17) | 30 (26 - 34) |
| Lebanon | 7121 (4762 - 9812) | 6151 (4142 - 8540) | 13272 (9808 - 17149) | 3801 (2487 - 5297) | 2720 (1788 - 3619) | 6521 (4869 - 8388) | 88 (58 - 123) | 69 (45 - 96) | 157 (117 - 203) |
| Libya | 4015 (3283 - 4870) | 3631 (2967 - 4471) | 7646 (6640 - 8813) | 2594 (2091 - 3170) | 1821 (1477 - 2239) | 4415 (3776 - 5114) | 69 (56 - 85) | 55 (44 - 67) | 124 (106 - 144) |
| Morocco | 22212 (15240 - 32098) | 31158 (20039 - 44708) | 53370 (40360 - 69302) | 16287 (11114 - 23790) | 14850 (9775 - 20744) | 31137 (24261 - 40882) | 416 (279 - 605) | 416 (271 - 592) | 832 (640 - 1096) |
| Oman | 1556 (1238 - 1907) | 968 (789 - 1212) | 2524 (2128 - 2896) | 785 (621 - 950) | 406 (326 - 494) | 1191 (990 - 1380) | 25 (20 - 31) | 13 (10 - 16) | 38 (32 - 44) |
| Pakistan | 91425 (75017 - 112765) | 162817 (123959 - 203775) | 254242 (209689 - 302397) | 60154 (49966 - 73543) | 67871 (51845 - 84324) | 128025 (109302 - 148317) | 1921 (1578 - 2377) | 2271 (1732 - 2805) | 4192 (3572 - 4851) |
| Palestine | 1595 (1201 - 2079) | 1885 (1429 - 2561) | 3479 (2832 - 4299) | 1080 (804 - 1401) | 778 (595 - 1035) | 1858 (1496 - 2250) | 38 (28 - 49) | 29 (23 - 39) | 66 (54 - 81) |
| Qatar | 711 (528 - 925) | 579 (415 - 763) | 1290 (1034 - 1592) | 333 (243 - 436) | 188 (137 - 243) | 521 (417 - 647) | 11 (8 - 14) | 7 (5 - 9) | 18 (15 - 22) |
| Saudi Arabia | 8642 (6974 - 10653) | 7084 (5597 - 8743) | 15726 (13455 - 18378) | 4725 (4167 - 5473) | 2868 (2545 - 3254) | 7593 (6918 - 8414) | 137 (120 - 158) | 88 (78 - 101) | 225 (204 - 249) |
| Somalia | 3146 (1338 - 7038) | 6716 (1927 - 16808) | 9862 (3353 - 23475) | 2637 (1052 - 5953) | 4028 (1047 - 9766) | 6665 (2126 - 15423) | 90 (38 - 204) | 139 (40 - 342) | 229 (83 - 550) |
| Sudan | 12225 (8591 - 17223) | 17516 (10194 - 26734) | 29740 (21475 - 39894) | 8953 (6214 - 12953) | 8194 (4977 - 11868) | 17147 (12710 - 22510) | 275 (189 - 397) | 270 (166 - 392) | 545 (400 - 719) |
| Syria | 4972 (4048 - 5934) | 5984 (4907 - 7266) | 10956 (9550 - 12553) | 3308 (2664 - 3949) | 2800 (2290 - 3299) | 6108 (5248 - 6932) | 94 (77 - 110) | 85 (70 - 100) | 178 (156 - 202) |
| Tunisia | 10348 (7767 - 13427) | 9122 (6502 - 11955) | 19471 (15273 - 23710) | 7222 (5410 - 9469) | 4048 (2902 - 5333) | 11270 (8806 - 13792) | 172 (129 - 228) | 106 (77 - 141) | 278 (219 - 338) |
| United Arab Emirates | 6197 (4044 - 9078) | 3050 (1989 - 4344) | 9247 (6512 - 12498) | 2314 (1554 - 3186) | 869 (610 - 1218) | 3183 (2291 - 4151) | 85 (56 - 118) | 35 (25 - 49) | 121 (86 - 158) |
| Yemen | 6951 (4439 - 10955) | 12999 (7153 - 23801) | 19950 (11767 - 33838) | 5146 (3186 - 8183) | 5895 (3303 - 9828) | 11040 (6416 - 17741) | 164 (101 - 261) | 202 (117 - 337) | 365 (217 - 591) |

| **eTable 3. Decomposition Analysis of Cancer Incidence by Cancer Type in the Eastern Mediterranean Region, both sexes, 2005 to 2015. (Global Burden of Disease Study 2015, Eastern Mediterranean Region, 2005-2015)** | | | | | | | | |
| --- | --- | --- | --- | --- | --- | --- | --- | --- |
| **Cause** | **Number of incident Cases** | | **Expected number of cases in 2015** | | **Change in incident cases 2005 to 2015 in %** | | | **Overall Change in %** |
|  | **2005** | **2015** | **Given population growth alone** | **Given population growth and aging** | **due to population growth** | **due to population ageing** | **due to change in incidence rates** |  |
| **All Cancers Groups** | 494690 | 722646 | 609771 | 670386 | 23.3 | 12.3 | 10.6 | 46.1 |
| **Lip and oral cavity cancer** | 24122 | 27021 | 29733 | 33237 | 23.3 | 14.5 | -25.8 | 12.0 |
| **Nasopharynx cancer** | 3846 | 5056 | 4740 | 5244 | 23.3 | 13.1 | -4.9 | 31.5 |
| **Other pharynx cancer** | 6083 | 7988 | 7499 | 8373 | 23.3 | 14.4 | -6.3 | 31.3 |
| **Esophageal cancer** | 12972 | 16788 | 15990 | 17761 | 23.3 | 13.7 | -7.5 | 29.4 |
| **Stomach cancer** | 34363 | 44818 | 42357 | 46725 | 23.3 | 12.7 | -5.5 | 30.4 |
| **Colon and rectum cancer** | 22485 | 35813 | 27716 | 30689 | 23.3 | 13.2 | 22.8 | 59.3 |
| **Liver cancer** | 18198 | 24569 | 22431 | 24869 | 23.3 | 13.4 | -1.6 | 35.0 |
| **Gallbladder cancer** | 5304 | 6926 | 6538 | 7238 | 23.3 | 13.2 | -5.9 | 30.6 |
| **Pancreatic cancer** | 7753 | 11168 | 9556 | 10559 | 23.3 | 12.9 | 7.9 | 44.1 |
| **Larynx cancer** | 11003 | 14863 | 13562 | 15150 | 23.3 | 14.4 | -2.6 | 35.1 |
| **Tracheal, Bronchial and Lung Cancer** | 33549 | 49530 | 41354 | 45791 | 23.3 | 13.2 | 11.1 | 47.6 |
| **Malignant skin melanoma** | 3552 | 5755 | 4378 | 4825 | 23.3 | 12.6 | 26.2 | 62.0 |
| **Non-melanoma skin cancer** | 9019 | 14057 | 11117 | 12258 | 23.3 | 12.7 | 19.9 | 55.9 |
| **Breast cancer** | 113843 | 179448 | 140327 | 157842 | 23.3 | 15.4 | 19.0 | 57.6 |
| **Cervical cancer** | 17344 | 19634 | 21379 | 23985 | 23.3 | 15.0 | -25.1 | 13.2 |
| **Uterine cancer** | 9350 | 14337 | 11525 | 12888 | 23.3 | 14.6 | 15.5 | 53.3 |
| **Ovarian cancer** | 7270 | 10946 | 8961 | 9783 | 23.3 | 11.3 | 16.0 | 50.6 |
| **Prostate cancer** | 16014 | 27534 | 19739 | 21220 | 23.3 | 9.2 | 39.4 | 71.9 |
| **Testicular cancer** | 1664 | 3144 | 2051 | 2192 | 23.3 | 8.4 | 57.2 | 88.9 |
| **Kidney cancer** | 5070 | 8321 | 6250 | 6789 | 23.3 | 10.6 | 30.2 | 64.1 |
| **Bladder cancer** | 20922 | 29854 | 25789 | 28628 | 23.3 | 13.6 | 5.9 | 42.7 |
| **Brain and nervous system cancer** | 16957 | 23851 | 20901 | 21974 | 23.3 | 6.3 | 11.1 | 40.7 |
| **Thyroid cancer** | 6672 | 11191 | 8224 | 9201 | 23.3 | 14.6 | 29.8 | 67.7 |
| **Mesothelioma** | 914 | 1100 | 1127 | 1254 | 23.3 | 14.0 | -16.9 | 20.3 |
| **Hodgkin lymphoma** | 4283 | 5620 | 5280 | 5453 | 23.3 | 4.0 | 3.9 | 31.2 |
| **Non-Hodgkin lymphoma** | 19912 | 31367 | 24544 | 26007 | 23.3 | 7.3 | 26.9 | 57.5 |
| **Multiple myeloma** | 3502 | 5336 | 4316 | 4792 | 23.3 | 13.6 | 15.6 | 52.4 |
| **Leukemia** | 34454 | 47680 | 42469 | 44585 | 23.3 | 6.1 | 9.0 | 38.4 |
| **Other neoplasms** | 26678 | 36523 | 32884 | 34425 | 23.3 | 5.8 | 7.9 | 36.9 |

| eTable 4: Comparison in total cancer incidence and mortality cases between Globocan 2012 (year 2012) and GBD 2015 (year 2015). (Global Burden of Disease Study 2015, Eastern Mediterranean Countries, 2015) | | | | | | | | |
| --- | --- | --- | --- | --- | --- | --- | --- | --- |
| Location | Incidence Globocan 2012 | Mortality Globocan 2012 | Incidence GBD 2015 | 95% UI GBD incidence | Mortality GBD 2015 | 95% UI GBD mortality | % Difference GBD Globocan (incidence) | % Difference GBD Globocan (mortality) |
| EMR | 546,630 | 360,814 | 722,646 | (660722 - 790102) | 379,091 | (350252 – 408579) | 32% | 5% |
| Afghanistan | 19,978 | 15,443 | 36,809 | (20719 - 62375) | 19,960 | (12339 - 28796) | 84% | 29% |
| Bahrain | 898 | 348 | 1,105 | (924 - 1326) | 476 | (399 - 565) | 23% | 37% |
| Djibouti | 581 | 435 | 1,153 | (600 - 2213) | 715 | (363 - 1364) | 98% | 64% |
| Egypt | 108,611 | 72,300 | 87,853 | (82803 - 94912) | 47,394 | (45414 - 50343) | -19% | -34% |
| Iraq | 25,666 | 17,460 | 41,208 | (31452 - 53209) | 20,793 | (16441 - 26207) | 61% | 19% |
| Iran | 84,829 | 53,350 | 95,011 | (79386 - 113095) | 49,179 | (40814 - 58144) | 12% | -8% |
| Jordan | 6,383 | 3,777 | 6,188 | (5407 - 7089) | 2,978 | (2633 - 3342) | -3% | -21% |
| Kuwait | 1,689 | 796 | 2,544 | (2236 - 2935) | 922 | (814 - 1059) | 51% | 16% |
| Lebanon | 9,059 | 4,831 | 13,272 | (9808 - 17149) | 6,521 | (4869 - 8388) | 47% | 35% |
| Libya | 6,077 | 3,527 | 7,646 | (6640 - 8813) | 4,415 | (3776 - 5114) | 26% | 25% |
| Morocco | 35,018 | 22,798 | 53,370 | (40360 - 69302) | 31,137 | (24261 - 40882) | 52% | 37% |
| Oman | 1,484 | 888 | 2,524 | (2128 - 2896) | 1,191 | (990 - 1380) | 70% | 34% |
| Pakistan | 148,041 | 101,113 | 254,242 | (209689 - 302397) | 128,025 | (109302 - 148317) | 72% | 27% |
| Qatar | 1,017 | 484 | 1,290 | (1034 - 1592) | 521 | (417 - 647) | 27% | 8% |
| Saudi Arabia | 17,522 | 9,134 | 15,726 | (13455 - 18378) | 7,593 | (6918 - 8414) | -10% | -17% |
| Somalia | 7,689 | 5,491 | 9,862 | (3353 - 23475) | 6,665 | (2126 - 15423) | 28% | 21% |
| Palestine | 3,464 | 2,180 | 3,479 | (2832 - 4299) | 1,858 | (1496 - 2250) | 0% | -15% |
| Sudan | 20,355 | 15,510 | 29,740 | (21475 - 39894) | 17,147 | (12710 - 22510) | 46% | 11% |
| Syria | 21,791 | 13,931 | 10,956 | (9550 - 12553) | 6,108 | (5248 - 6932) | -50% | -56% |
| Tunisia | 12,189 | 7,339 | 19,471 | (15273 - 23710) | 11,270 | (8806 - 13792) | 60% | 54% |
| United Arab Emirates | 2,935 | 1,257 | 9,247 | (6512 - 12498) | 3,183 | (2291 - 4151) | 215% | 153% |
| Yemen | 11,354 | 8,422 | 19,950 | (11767 - 33838) | 11,040 | (6416 - 17741) | 76% | 31% |

eFigure 1 Contribution of Years Lived with Disability and Years of Life Lost to Disability Adjusted Life Years by cancer in the Eastern Mediterranean Region, both sexes, 2015. (Global Burden of Disease Study 2015, Eastern Mediterranean Region, 2015)

YLL: Years of Life Lost, YLD: Years Lived with Disability, DALY: Disability Adjusted Life Years
